# Multi-scale temporal patterns in fish presence in a high-velocity tidal channel

**DOI:** 10.1371/journal.pone.0176405

**Published:** 2017-05-11

**Authors:** Haley A. Viehman, Gayle Barbin Zydlewski

**Affiliations:** School of Marine Sciences, University of Maine, Orono, Maine, United States of America; University of Waikato, NEW ZEALAND

## Abstract

The natural variation of fish presence in high-velocity tidal channels is not well understood. A better understanding of fish use of these areas would aid in predicting fish interactions with marine hydrokinetic (MHK) devices, the effects of which are uncertain but of high concern. To characterize the patterns in fish presence at a tidal energy site in Cobscook Bay, Maine, we examined two years of hydroacoustic data continuously collected at the proposed depth of an MHK turbine with a bottom-mounted, side-looking echosounder. The maximum number of fish counted per hour ranged from hundreds in the early spring to over 1,000 in the fall. Counts varied greatly with tidal and diel cycles in a seasonally changing relationship, likely linked to the seasonally changing fish community of the bay. In the winter and spring, higher hourly counts were generally confined to ebb tides and low slack tides near sunrise and sunset. In summer and fall of each year, the highest fish counts shifted to night and occurred during ebb, low slack, and flood tides. Fish counts were not linked to current speed, and did not decrease as current speed increased, contrary to observations at other tidal power sites. As fish counts may be proportional to the encounter rate of fish with an MHK turbine at the same depth, highly variable counts indicate that the risk to fish is similarly variable. The links between fish presence and environmental cycles at this site will likely be present at other locations with similar environmental forcing, making these observations useful in predicting potential fish interactions at tidal energy sites worldwide.

## Introduction

Relatively little is known about how fish use areas with fast tidal currents. Fish activity levels and movement patterns vary on a wide range of spatial and temporal scales over the course of their lives, often in ways that are species- and life-stage specific [1–3]. Many fish movements are related to environmental changes; for example, vertical migrations linked to the diel cycle, tidal movements into the intertidal zone, or seasonal movements on- or off-shore [1, 3]. In the high-velocity channels targeted for tidal energy extraction, underwater conditions change rapidly and fish presence and distribution likely fluctuates with similar magnitude and frequency. For example, there is already well-established evidence of some fish species changing their location in the water column to take advantage of favorable tidal currents in on- or off-shore migrations, a behavior known as selective tidal stream transport (see reviews [3, 4]). However, many studies of this behavior did not collect data with sufficient resolution and duration to fully describe the wide-ranging scales of cyclic temporal variation in fish presence in these high-flow tidal environments.

A study that aims to describe temporal patterns in any metric of interest would include sampling with high resolution for a long time. High-resolution sampling would occur at several times the frequency of the shortest cycle present, and long-duration sampling would span several times the duration of the longest cycle present [5]. However, studies of biological systems are more typically designed to focus on either high-resolution or long-duration, rather than both [6], due to cost or gear limitations. Many high-resolution studies have occurred over short periods of time [7–13]. Some have sought to characterize both short- and long-term variability by carrying out multiple short-term, high-resolution surveys spaced over a long period of time [14, 15]. However, when focusing on frequency or duration, prior assumptions about cyclical patterns present in a variable of interest can influence study design and can restrict the scope of results. For example, if fish are assumed to follow a diel migration pattern, when in reality a diel and tidal cycles both influence their distribution, averaging data by day and night can alias the results [12].

Long-term, high-resolution sampling is less common but ideal for understanding biological processes at sites where large changes may occur over multiple, wide-ranging time scales. One method of sampling large volumes of water rapidly for long periods of time is stationary hydroacoustics. Long-term stationary echosounder deployments have been used to examine the temporal variability of different biological sound-scatterers in the ocean, including phytoplankton and zooplankton [16], zooplankton [17, 18], and zooplankton and fish [6, 19]. These studies all found the presence and vertical distribution of their pelagic subjects to be linked to changes in their physical environment on a variety of time scales, including tidal, diel, and/or seasonal cycles, and these patterns were generally not constant over the duration of sampling time.

Transient cycles are a common characteristic of biological processes, and wavelet analysis is an effective tool for detecting and describing such patterns [20]. Wavelet analysis works by simultaneously decomposing time series data across both the frequency and time domains. This technique has an advantage over temporal analysis methods, such as spectral analysis via the Fourier transform [6, 13] or autocorrelation functions [12], because it does not assume the time series to be unchanging (stationary) over time [20, 21]. Blauw et al. [22] used wavelets to explore the patterns present in their time series related to phytoplankton in the North Sea, as well as to relate phytoplankton growth with the tidal cycle and suspended particulate matter. Urmy et al. [6] applied wavelets in part of their assessment of changing patterns in nekton density and vertical distribution in Monterey Bay. Apart from Urmy et al. [6], the use of wavelets in long-term studies of marine fish has been primarily to assess patterns in yearly fishery catch rates and how they relate to climatic oscillations, which does not address the higher-frequency cycles pertinent to a tidal channel [23, 24].

In this study, we used wavelet analysis to describe the temporal variation in fish passage rate in Cobscook Bay, Maine. Data collection occurred at a site evaluated for tidal energy development, where an echosounder had been installed on the seafloor to monitor fish encounter with a marine hydrokinetic (MHK) turbine. After the turbine’s removal, the echosounder continued to operate, and we detected fish in 2 years of high-resolution hydroacoustic data collected at turbine depth. Our goals were to obtain a better understanding of fish presence in this portion of the water column of this high-speed tidal channel, and to consider the implications of our findings for potential turbine effects.

## Methods

### Data collection

**Fig 1:**
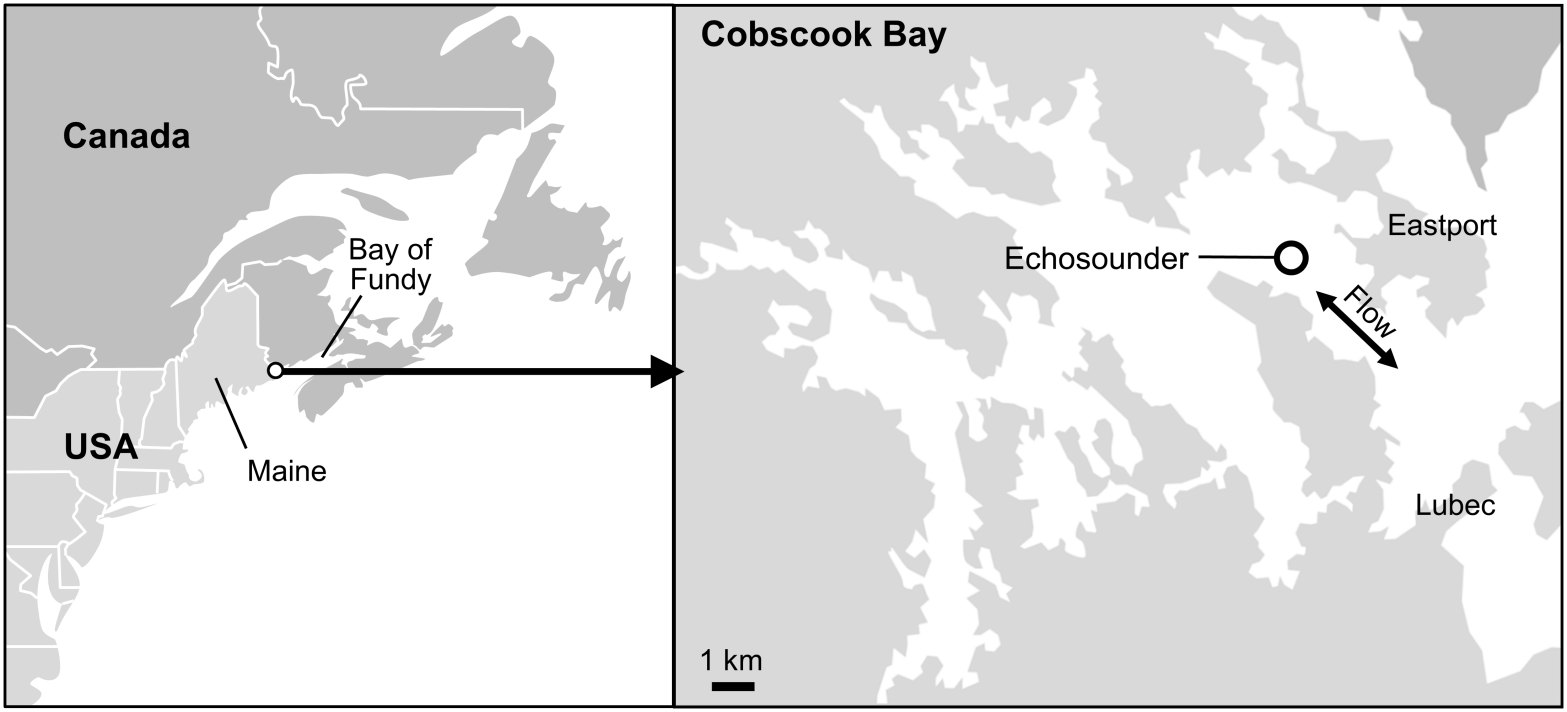
Map of study area with location of echosounder.

**Fig 2:**
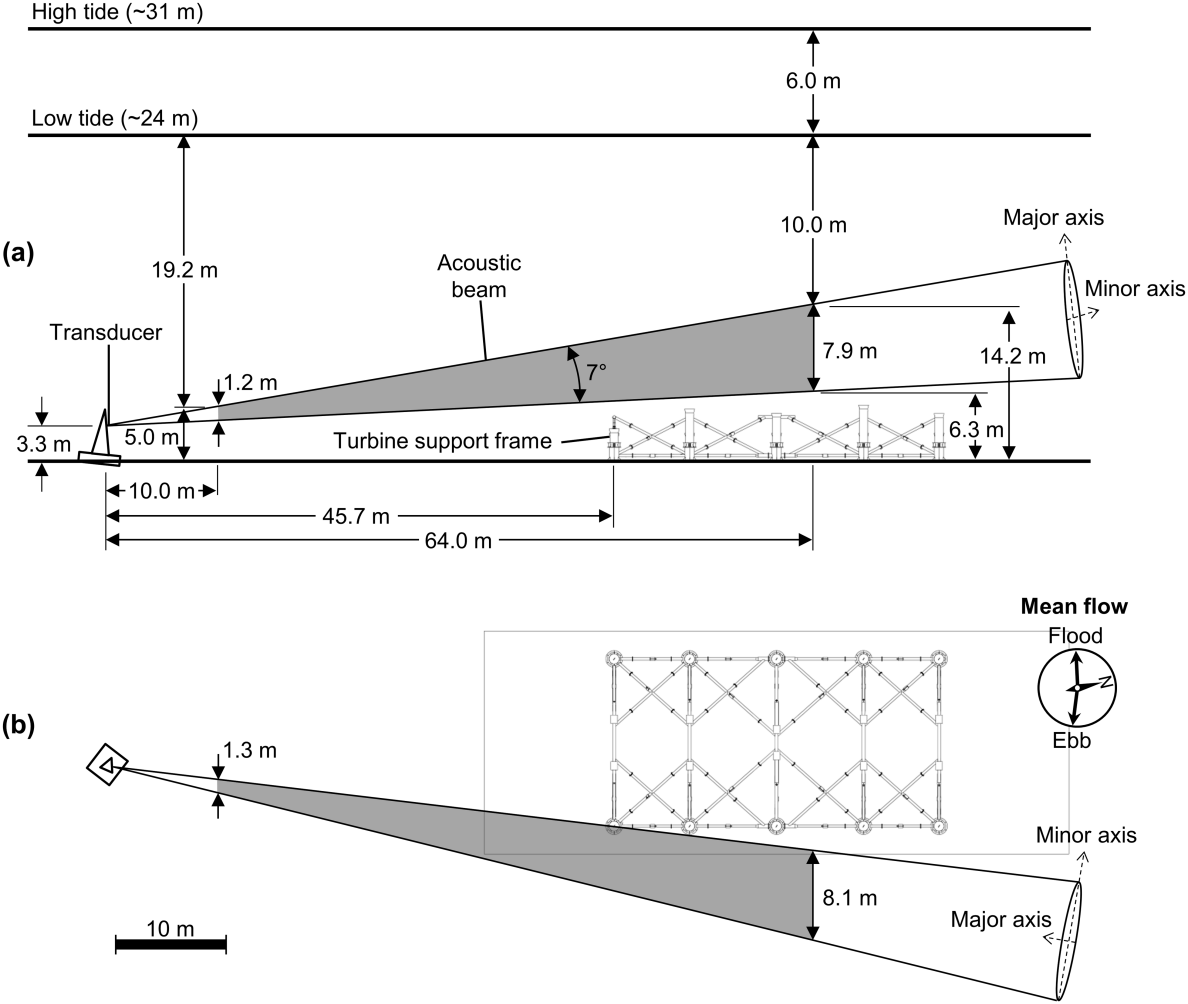
Echosounder setup in Cobscook Bay, Maine. The bottom support frame of the MHK tidal energy device was present during data collection. The gray area indicates the sampled volume used in this study. Device schematic provided by Ocean Renewable Power Company.

Hydroacoustic data were collected at a tidal energy site in Cobscook Bay, ME, from July 15, 2013, to July 28, 2015 (Fig 1). The site was located in outer Cobscook Bay, where current speed ranges from 0 to approximately 2 m·s^-1^, depending on the tidal and lunar phase [14, 25, 26]. The turbine and echosounder were located mid-channel in a shallow area (24 to 31 m depth from low to high tide) between two deeper areas (~50 m maximum depth) to the northeast and southwest. The echosounder was installed by Ocean Renewable Power Company (ORPC) to observe fish behavior near its MHK device, which was deployed at the site from August 2012 to July 2013. The use of this location by ORPC was authorized by the Federal Energy Regulatory Commission (FERC), the Maine Department of Environmental Protection’s Maine Waterway Development and Conservation Act General Permit and Water Quality Certification (Eastport, #L-25468-35-A-N), and the Maine Department of Conservation's Submerged Land Lease (#1684). The transducer was oriented to sample the area along the face of the turbine (Fig 2), and after the turbine was removed in July 2013, the echosounder continued to collect data. The Simrad EK60 echosounder used a 200 kHz, 7° split beam transducer, which was mounted 3.4 m above the sea floor and 44.5 m from the turbine’s support frame and angled 6.2° above horizontal. The echosounder sampled an approximately conical volume of water 5 times (pings) per second, using a pulse duration of 0.256 ms and transmit power of 120 W. The current flowed approximately perpendicular to the sampled volume. Most fish moved with the current and were therefore detected by several sequential pings as they passed through the acoustic beam, even at peak flows.

**Fig 3:**
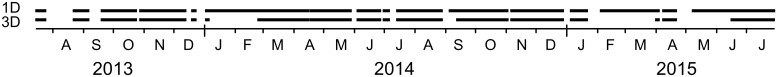
Periods of continuous data collection. 1D denotes data which did not always include angular information, which were used for obtaining fish counts; 3D denotes data which did include angular information, which were used for determining fish target strength and times of slack tide (description in text).

The echosounder operated nearly continuously for the two years it was deployed, but there were several gaps in data collection due to technical issues or necessary shut-down of the echosounder during turbine-related activities, such as diver inspection (Fig 3). The final dataset included 582 days of data. However, not all of these data were complete: at times, the angular information associated with acoustic targets was not returned. Major and minor axis angles describe a target’s position within the beam’s cross-section. Absence of these data limited positioning of acoustic targets to 1 dimension (range), rather than 3 (range, minor-axis angle, and major-axis angle) (Fig 3). This issue was usually resolved by restarting the system, but the manufacturer was unable to identify the cause.

### Data processing

Acoustic data were processed using Echoview^®^ software (6.1, Myriax, Hobart, Australia). Processing consisted of noise removal, fish tracking, and fish track export. Fish tracking was carried out both in 2D (which uses time and range of acoustic targets to detect fish) and 4D (which uses time, range, and major and minor axis angles). 4D tracking could only be carried out when 3D information was available (Fig 3), which left large gaps in the fish-track dataset. 2D tracking was therefore used to generate the time series of hourly fish counts, as range and time information were returned whenever the echosounder was running, leaving fewer and smaller gaps. When angular data were available, fish were tracked in 4D to obtain accurate measures of fish target strength and to verify fish counts supplied by 2D tracking. 4D tracking also provided the directions traveled by fish. These directions were used to accurately model the tidal cycle for the duration of data collection, as the nearest tidal station is outside of Cobscook Bay and provides tide times that differ substantially from what is observed within the bay.

#### Noise removal

**Fig 4:**
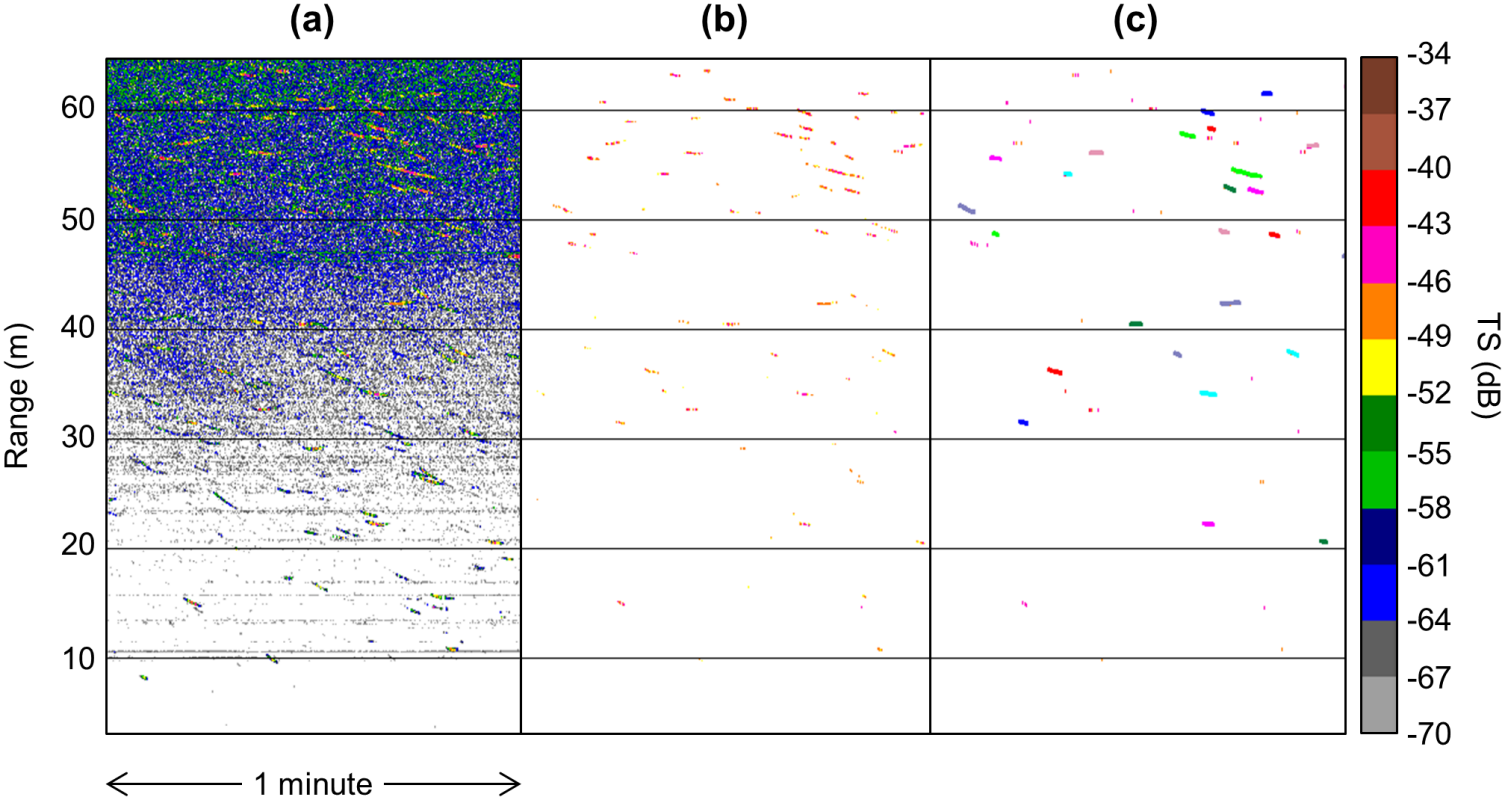
Acoustic data processing example. (a) Raw target strength data (scale in dB to right) showing multiple fish tracks and background noise. (b) Target strength data with noise removed and -50 dB TS threshold applied. (c) Single targets detected from cleaned target strength data with fish tracks (colored lines) overlaid.

The acoustic data included several types signal that were not from individual fish, which had to be removed before fish could be tracked (Fig 4). This signal included small, non-fish targets (e.g., large zooplankton), interference from the surface and entrained air near the surface, and schools of fish (in which individual fish cannot be accurately tracked). Target strength (TS) is a measure of the proportion of sound energy that is reflected back to the transducer by an object, and it is roughly proportional to the object’s size [27]. Applying a TS threshold of -50 dB eliminated most signal from small, non-fish targets. Using an equation for side-aspect fish TS to length developed by Love [28], this threshold roughly equates to a fish length of 4 cm, though fish TS varies greatly with anatomy and orientation relative to the beam. Surface interference was removed by limiting the maximum analysis range to 64 m, which is the range at which entrained air from the surface began heavily interfering with the acoustic signal. Sound attenuates the farther it travels, resulting in a decreasing signal-to-noise ratio with distance from the transducer. This was visible in the acoustic data as a gradient of ‘background noise’ (Fig 4a), the intensity of which varied over time with water height and weather conditions. This type of gradually changing noise was removed using the method developed by De Robertis and Higginbottom [29] for volume backscatter data, modified to apply to our TS data. This method allows background noise estimates to vary over time and removes acoustic signal with low signal-to-noise ratio. The method worked well to remove the gradually fluctuating background noise in our TS data. Intermittent acoustic signal from other sources, such as schools and clouds of entrained air, was removed using multiple resampling and masking steps with Echoview^®^ virtual operators. Schools of fish were generally indistinguishable from entrained air, so were omitted from analysis. These steps were worked into a template that was then applied to all data using Echoview^®^’s scripting module.

#### Fish tracking

Once unwanted acoustic signal was removed from the acoustic data, single targets were detected and fish were tracked using both split beam (4D) and single beam (2D) methods (Fig 4c). As mentioned above, both tracking methods were used because of the intermittent angular data. Split beam single target detection and 4D-fish tracking rely on these angular data to determine if each echo was caused by a single object or multiple, and to correct TS values for beam pattern effects. Single beam single target detection and 2D fish tracking methods do not incorporate angular data. While this can result in less accurate fish tracking and TS estimates, in this case it provided a more complete time series of fish counts. Fish tracked via single beam, 2D methods were therefore used to construct the time series of passage rate, and split-beam, 4D methods were used to supplement our interpretation of this time series, providing estimates of fish TS and fish swimming direction (as an indicator of tidal stage), and verifying temporal patterns in the time series made from fish tracked in 2D. (Table 1)

**Table 1:** Echoview^®^ acoustic data processing parameters.

Process	Parameter	1D data	3D data
Single target detection	TS threshold	-50 dB	-50 dB
	Pulse length determination level	6.00 dB	6.00 dB
	Min. normalized pulse length	0.50	0.20
	Max. normalized pulse length	2.00	2.00
	Beam compensation model		Simrad LOBE
	Max. beam compensation		12.00 dB
	Max. standard deviation of angles		0.5°
	Angle range		-2.5° to 2.5°
Fish tracking	Data	2D	4D
	Alpha (range)	0.8	0.7, 0.7, 0.8
	Beta (range)	0.5	0.5, 0.5, 0.5
	Exclusion distance (range)	0.5 m	1.5, 1.5, 0.5 m
	Missed ping expansion (range)	0%	0%
	Weights	0	0
	Min. number of single targets in a track	5	5
	Min. number of pings in a track	5 pings	5 pings
	Max. gap between single targets	3 pings	3 pings

All tracks were exported for further analysis in R (3.1.1, R Core Team, Vienna, Austria).

### Data analysis

#### Fish target strength

The TS of fish tracked with 4D methods was summarized with median and interquartile range, but closer examination was avoided due to the mixed nature of the fish assemblage in Cobscook Bay and our inability to identify the species of each detected fish.

#### Wavelet transform

The time associated with each tracked fish was used to count fish in 1-hr time increments, producing a time series of the number of fish to pass through the sampled volume with 1-hr resolution. A wavelet transform was then used to inspect patterns in hourly counts and how they changed over time. Wavelet transforms simultaneously decompose time series data across both the frequency and time domains by convolving the time series with a wave form (the ‘wavelet’), which is scaled up or down within a chosen range as it is moved along the time axis. The result is a wavelet spectrum: a 2D representation of the power of each periodic signal that composes the original time series, across the time spanned by the dataset. We applied a continuous wavelet transform using the Morlet wavelet to the 2-yr time series of hourly fish counts (package *WaveletComp* in R [30]). The maximum frequency included was 2 times the sampling frequency (a period of 2 hours), as this would be the highest possible frequency that could theoretically be characterized (i.e., the Nyquist frequency). The maximum period was limited to one-half of the total sampling period (1 year) because larger periods are overwhelmed by edge effects. The wavelet spectrum was tested for significance against a theoretical spectrum of white noise (i.e., a random signal), simulated 100 times, with a significance level of 0.05 (function *analyze*.*wavelet* in *WaveletComp*).

#### Gap-filling

The wavelet transform requires a time series with no missing values, so data gaps (Fig 3a) were filled with new values which would minimally impact the resulting wavelet spectrum. The effects of several gap-filling methods on results were examined using a simulated time series (see S1a Fig) composed of known periodic components. This known time series allowed a comparison of its true wavelet spectrum to those that resulted from different gap-filling procedures (S1b Fig). The hourly fish count time series we ultimately wanted to analyze contained 20 gaps ranging from 2 hr to 29.5 days, separated by 1 to 57 days of continuous data (Figs 3 and 1D). These same gaps were created in the simulated data set (S1a Fig). Simply filling these gaps with a median value or linear interpolation introduced intermittent noise to the resulting wavelet spectrum, significantly increasing the power contained within periods of 4–20 days and 40–100 days (S1c Fig). These effects were reduced by filling gaps with a periodic signal (S1d Fig). The individual sinusoidal components of the periodic signal were chosen to be characteristic of the time series as a whole, with periods determined using a Fourier transform of the median-filled time series. A linear model of the form

$$y=\;\sum_{i=1}^{N}A_{i}\text{sin}\left(\frac{2\pi }{P_{i}}t\right)+B_{i}\text{cos}\left(\frac{2\pi }{P_{i}}t\right)+\varepsilon$$

was fit to the data surrounding each gap, where y is hourly fish count, P_i_ is the i^th^ period of the above set, and A_i_ and B_i_ are amplitudes obtained by minimizing error ε. Each model was fit using a subset of the time series that was within 1 gap-width or 48 hr to either side of a gap, whichever was longer. Short gaps did not benefit from the use of comparably long periods, so the longest period included in each gap model was limited to twice the length of subset used for fitting. Once the models were fit to data surrounding each gap, they were used to predict missing values. Random error with the same standard deviation as the model residuals was added to the predicted gap values, as this was found to slightly reduce the noise introduced to the wavelet spectrum by the gap-filling procedure (S1e Fig). Once the gap-filling method was verified on the simulated dataset, it was applied to the actual time series. The sinusoidal components used to fill the gaps had periods of 0.5, 1.0, 14.7, 27.6, 182, and 365 days.

#### Tidal stage modeling

**Fig 5:**
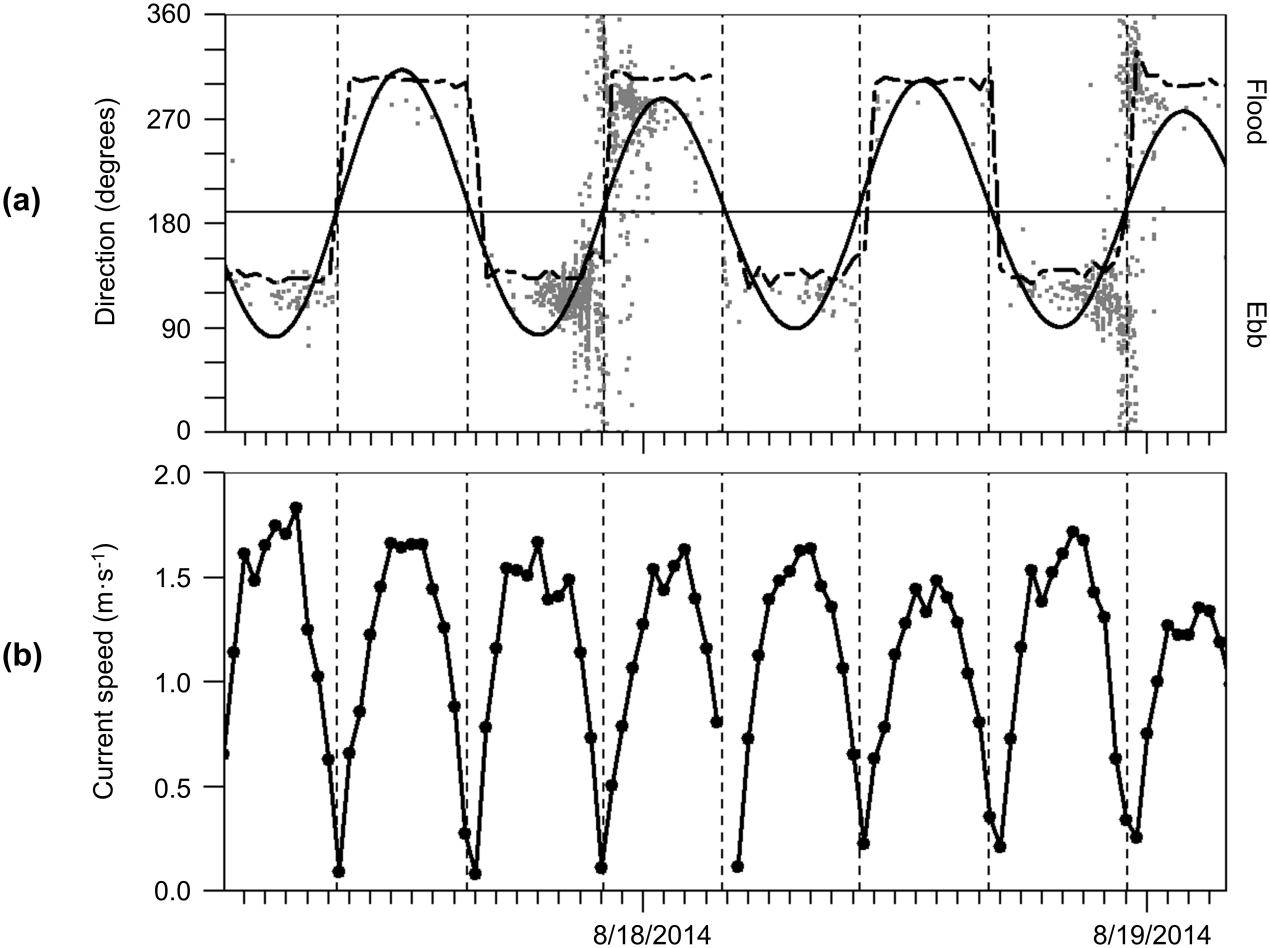
Fish movement direction with current speed and direction collected concurrently at the study site in August 2014. (a) Individual fish movement direction (gray points) shown with fitted tidal model (solid line) and its midline (horizontal line), which were used to estimate times of slack tide (vertical dashed lines). Current direction measured concurrently by an ADCP is overlaid (dashed line) (b) Current speed collected concurrently, with same times of slack tide as shown in a.

To examine patterns in fish presence with respect to the tidal stage, periods of low, high, ebb, and flood tides needed to be identified. However, the nearest tide station was outside of Cobscook Bay and did not accurately predict tide times at the study site. Only a few 24-hr spans of current velocity data were collected at this site during the study period, from a downward-facing Acoustic Doppler Current Profiler (ADCP) mounted vessel moored nearby as part of another study. Split beam (4D) fish tracking methods provided the direction traveled by individual fish. At this site, fish move almost exclusively in the direction of the current except at slack tides, when movement is more uniformly distributed for a brief period of time [31]. Fish direction could therefore be used as a proxy for tidal current direction, which indicated tidal stage. This correlation was verified by comparing fish direction and predicted tidal stages to the several instances of ADCP data that were available (Fig 5). Shifts in fish movement direction (Fig 5a) corresponded to slack tides as indicated by the ADCP data (Fig 5b). The square-wave pattern of fish movement direction was very similar to that of the measured current direction, and the transitions from one tidal stage to another occurred at the same times. Flood and ebb tide fish movement directions (approximately 285° and 120°, respectively) also aligned well with current direction modeled at a nearby location, presented by Xu et al. [25], and with other measurements of current direction at the study site (ORPC personal communication).

As large gaps existed in the 3D acoustic data, and therefore in the swimming directions available from 4D fish tracking, a tidal model was fit to existing fish direction data which could then be used to accurately predict tidal stage, even when 3D data were missing (thick solid line in Fig 5a). This tidal model was a summation of multiple sinusoids with varying periods, including the ten tidal components that have been used in shorter-term modeling studies of Cobscook Bay tidal currents (M2, K1, K2, N2, S2, O1, L2, M4, NU2, 2N2 [32]) and five longer-period tidal components that would be relevant to this 2-year span (P1, Q1, MF, MM, SSA). The model was constructed as for the gap-filling step, except in this case P_i_ was the i^th^ period of the 15 tidal components listed above. Amplitudes A_i_ and B_i_ were obtained by fitting a linear model (the same form as for gap-filling) to the available fish direction data from 4D fish tracking. The sinusoid peaks were defined as peak flood tides, the troughs were peak ebb tides, and slack tides (high or low) occurred wherever the sinusoid crossed its midline (Fig 5a).

## Results

### Target strength

The median target strength estimated from fish tracked using the 4D method was -45.1 dB, with an interquartile range of -46.5 to -43.4 dB. Using Love’s general side-aspect TS-length equation [28], this corresponds to a median fish length of roughly 6 cm, with an interquartile range of 5 to 7 cm.

### Time series

**Fig 6:**
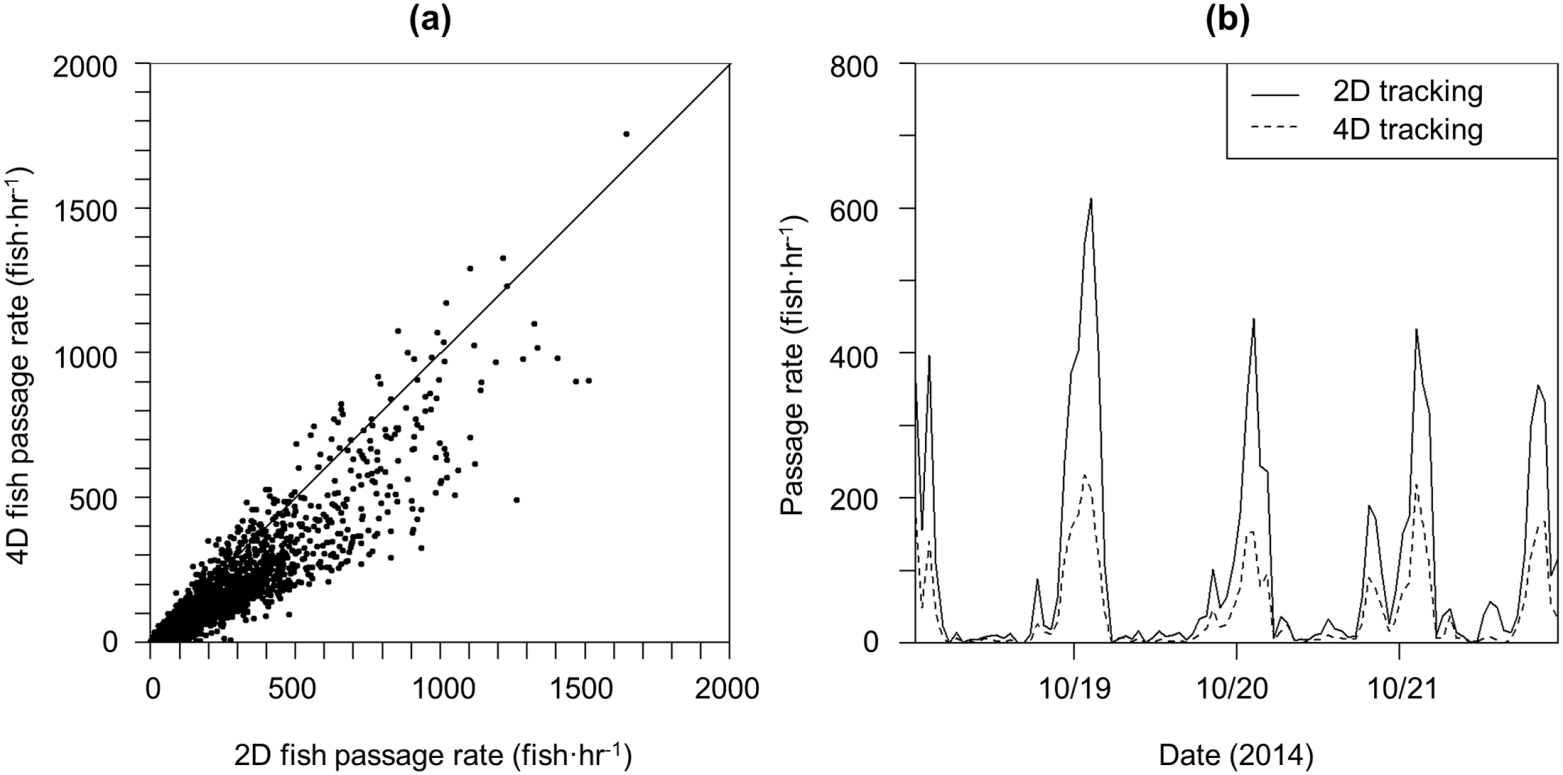
Comparison of hourly fish counts obtained via 2D and 4D tracking. (a) 4D vs. 2D counts, shown with 1:1 line. (b) Subset of 2D (solid line) and 4D (dashed line) counts from October 2014.

Hourly fish counts determined through 2D and 4D tracking methods were highly correlated with each other (Fig 6). Counts obtained via 2D methods were higher than those obtained using 4D methods due to the less stringent target quality requirements of 2D tracking (Fig 6a), but when data were available in both datasets, the patterns in hourly counts were the same (Fig 6b).

**Fig 7:**
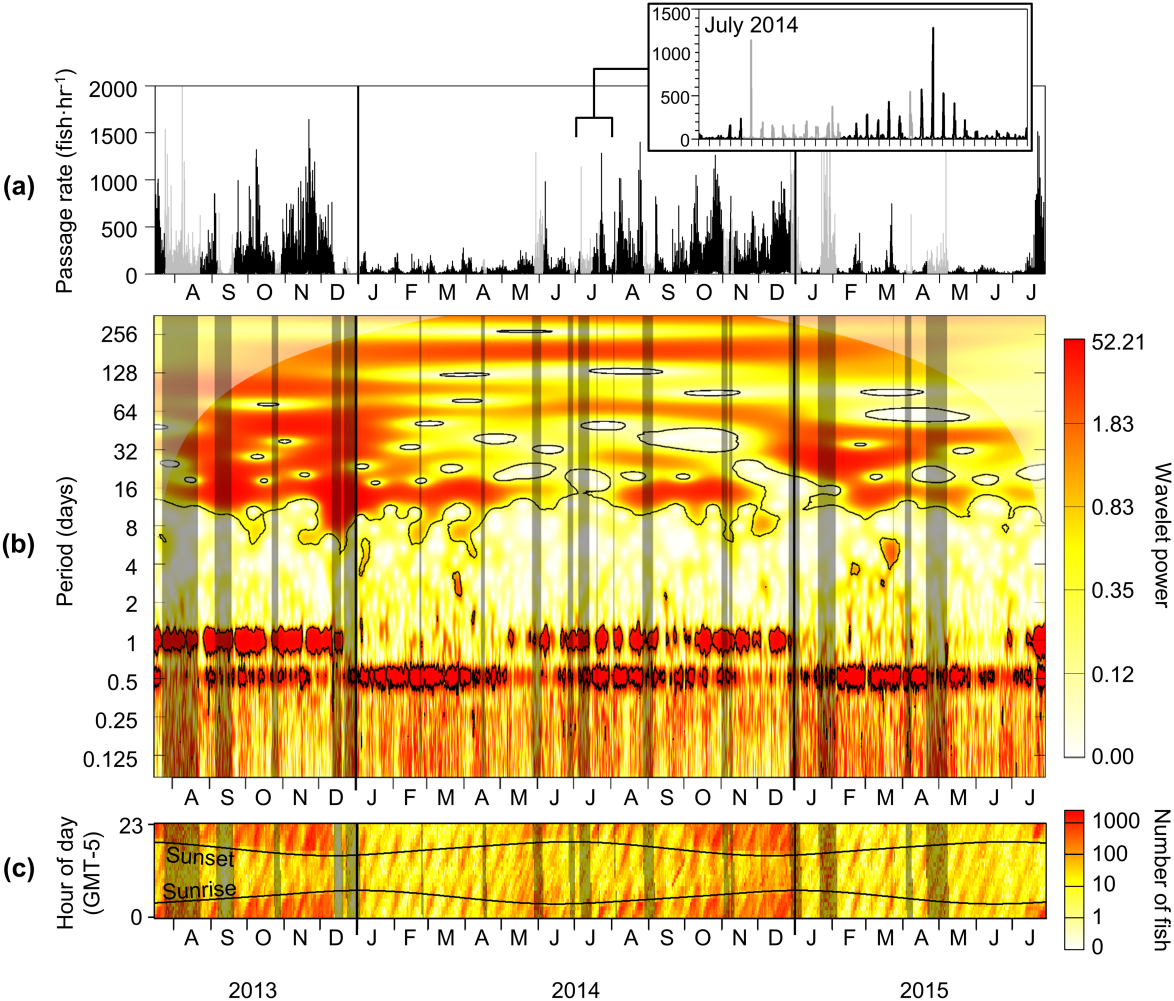
Wavelet transform of hourly fish counts. (a) Time series of hourly fish counts. Gaps in data that were filled using the method described in the text are shown in gray. Inset expands time series from July 2014. (b) Wavelet transform of log-transformed time series. Color indicates the magnitude of the wavelet power, with darker, redder colors indicating higher power. Black contours enclose areas of significance at the 5% significance level. The transparent white fill indicates the cone of influence, within which power values may be reduced by edge effects. Darkened rectangles indicate where gaps in the time series were filled. (c) Fish counts from each hour of each day of the time series, condensed here for easy comparison to a and b. An expanded version of c is shown in Fig 8. Darker, redder colors indicate higher fish counts. Horizontal curved lines indicate times of sunrise and sunset, and shaded rectangles indicate filled gaps in the time series.

Hourly fish counts varied greatly over the two years spanned by the dataset, on both short and long time scales (Fig 7a) [33]. Overall, counts were highest in late summer and fall of both years, reaching over 1,000 fish in a single hour. During winter and spring, count maxima were in the hundreds of fish. Hourly counts could increase from 0 to hundreds of fish in a matter of hours, and it was visually evident that this variation was cyclic in nature (Figs 6b and 7a). Tests of the gap-filling method on simulated data (S1 Fig) indicated that periodicities in the 40–100 day range could be influenced by the filled data values.

### Wavelet transform

The red horizontal band at the top of the wavelet transform (Fig 7b) indicated the presence of a 365-day periodicity, which is indicative of the seasonal cycle that was evident in the raw time series (Fig 7a). This seasonal change in hourly counts slightly lagged the seasonal change in temperature, with lowest counts between winter and spring (e.g., March) and highest in the fall (August through November). A continuous band in the wavelet transform at the 182-day periodicity and fainter, less continuous bands at approximately 14 and 28 days were also present, and coincided with tidal periodic components [32]. The intermittent areas of high power in the 40–100 day periodicities were likely artifacts related to the filled gaps in the time series (S1 Fig).

The strongest features of the wavelet transform were bands near 12- and 24-hr periods, which indicated variation in fish counts strongly related to the tidal (and/or semi-diel) and diel cycles. These two periodicities were not constant throughout the dataset but followed similar patterns in each year sampled, though with some variation in timing. The 24-hour periodicity was present only in the summer and fall, emerging in June in 2014 and 2015. The 12-hour periodicity was present throughout the year, but was most evident in the winter and spring (from January through May). For the rest of the year, the 12-hr periodicity was present but intermittent.

**Fig 8:**
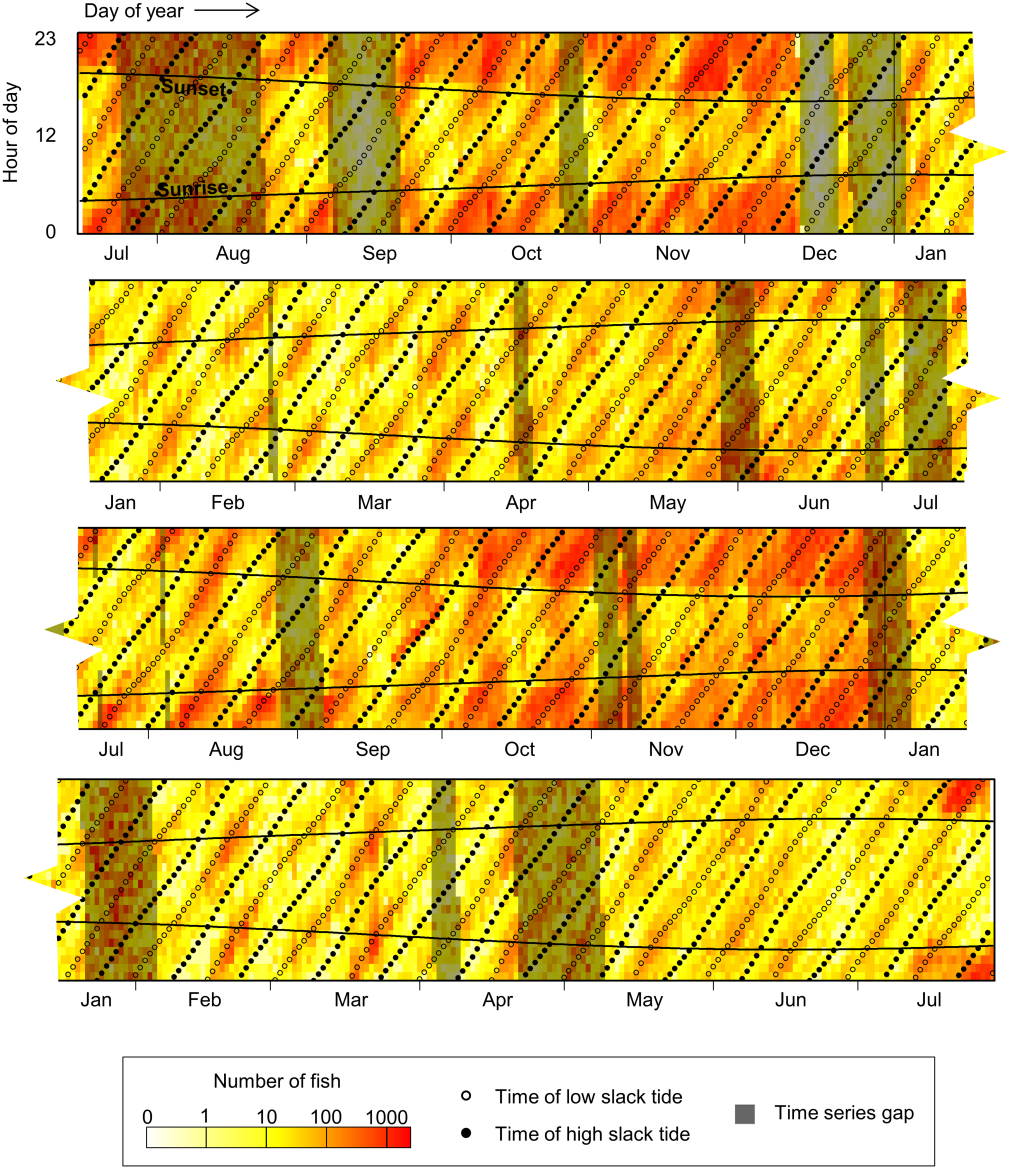
Hourly fish counts. Rows are each hour of the day, and columns are every day of the time series, which spans July 2013 to July 2015. Darker, redder colors indicate higher hourly fish counts. Gaps in the time series (filled using method described in text) are indicated by darkened rectangles. Horizontal curved lines indicate time of sunrise and sunset. Points indicate times of low (open circles) and high (solid circles) slack tides. A condensed version of this figure is shown in Fig 7c.

Visual inspection of fish counts at each hour of the day (Fig 7c) revealed obvious changes in counts that corresponded with the presence and absence of 12- and 24-hr periodicities in the wavelet transform. When viewed in relation to tidal and diel stage (Fig 8), it was clear that fish counts varied with tidal and diel cycles in a seasonally shifting relationship. In the winter and spring of each year (from January through May), when the 12-hr periodicity was present but not the 24-hr, the highest fish counts occurred on the second half of the ebb tide and at low tide, mainly near sunrise and sunset but with no strong difference between day and night. In the summer (June through August), when both the 12-and 24-hr periodicities were evident, the highest counts began shifting to night and were still mostly associated with low tide, though sometimes in the second half of ebb or first half of flood. In the fall and early winter (September through December), the 24-hr periodicity became more prominent while the 12-hr periodicity became less consistent. At this time, hourly fish counts were clearly higher at night than during the day, and the association with tidal stage was less clear, though counts were generally lower at high tide than at ebb, low, and flood tides. Between December and January, this pattern in fish counts quickly returned to peaks at low or ebb tides at sunrise and sunset.

## Discussion

This high-resolution, long-term time series indicated that fish presence was highly variable and strongly linked to multiple environmental cycles, including tidal, diel, and seasonal cycles. This is not surprising, given the high-magnitude seasonal changes (e.g. temperature and day length) and tidal forcing at this site, and that fish movements have been linked to such environmental changes in multiple studies (see reviews [1, 4]). The hourly fish count varied most with tidal and diel cycle, but the wavelet transform revealed that the relationship with these cycles was not constant over the course of the year, highlighting the utility of this temporal analysis method for ecological time series. Fish counts were linked strongly to the interaction of these two cycles in a relationship that varied with season. This adds to previous findings linking similarly interacting environmental cycles with fish presence and feeding in intertidal habitats [11, 34] and spatial distribution of fish and their predators and prey in tidal current systems [7, 9, 10].

All interactions of fish count with the tidal cycle were modified by the diel cycle. In the winter through spring, the highest hourly counts were confined to low slack and ebb tides, but within crepuscular periods. Higher counts shifted into night during the summer, expanding to flood and ebb tide in addition to low slack and remaining this way through the fall. These results are consistent with a previous study we carried out at this site, in which we conducted 24-hr surveys of fish density and vertical distribution using a downward-looking, vessel-mounted echosounder in March, May, June, September, and November 2011 [14]. In that study, we did not examine slack tides. However, we found that fish density was generally higher during the ebb tide than the flood tide in the first part of the year, and that this difference was less evident toward the end of the year. The vertical distribution of fish did not differ consistently between ebb and flood tide, so higher ebb- or flood-tide counts may be related to horizontal, rather than vertical, tidal fish movement. For example, fish may move between channel edge habitats and the middle of the channel based on flow direction, or be carried through different parts of the channel during ebb and flood tides on asymmetric flow paths. A tidal front created by the interaction of the currents and bathymetry is sometimes visible just south of the study site. Tidally-induced hydrodynamic features have been associated with higher fish concentrations at other locations [10, 35], and the formation or movement of this or other hydrodynamic structures at different stages of the tide could also affect when fish are detected at the site.

The diel differences in hourly fish counts which we observed, on the other hand, could have been related to changes in vertical fish distribution. Unlike tidal stage, our previous study of this site found consistent changes in fish vertical distribution related to diel stage: during the day, fish were more concentrated near the sea floor or surface, depending on time of year, but at night, fish spread out in the water column [14]. In that study, this difference was visually apparent in May, June, August, and September surveys, but not in March. In the present study, we sampled only the mid- or lower-water column (depending on water height). So, if the same diel shifts in vertical distribution were occurring during the present study period, from spring through fall most fish would have been outside of our sampled volume during the day but would have moved within view at night, and in the winter and early spring, there would be little day-night difference in hourly counts. This is consistent with our results, with higher fish counts occurring at night from June through December. However, the influence of diel cycle was not completely absent in the winter and early spring, as counts consistently peaked near dawn and dusk when those times coincided with low or ebbing tides. Our previous work only compared day- and night-averaged vertical distributions, so we had not captured any changes in fish activity related to dawn and dusk. By expecting and searching for 24-hr diel differences in fish vertical distribution, we did not detect this crepuscular increase in fish numbers that dominated for a large portion of the year. Prior assumptions such as this have likely influenced results in other studies of fish biology [12], highlighting the utility of long-term, high-resolution data.

It is important to remember that only individual fish were included in this study, as individuals within schools could not be separated from their neighbors, and fish schools often could not be distinguished from patches of entrained air. Fish in schools were therefore omitted, which potentially affected the patterns in hourly counts that we were able to measure. For example, we previously observed that many dense schools are present in Cobscook Bay in the spring [14, unpublished data], likely larval Atlantic herring (*Clupea harengus*) [15]. These schools contributed to high density indices in spring that were comparable to indices from the fall [14, unpublished data]. However, in the present study, hourly fish counts in the spring were much lower than in the fall, which could have been due to the exclusion of these schools. Individually, larval herring may not be strong enough acoustic targets to be tracked with a -50 dB TS threshold, so even if schools were to spread out (e.g., at night [12, 36]), they would be unlikely to contribute to fish counts. However, many schooling fish species have been observed to spread out in lower light levels, including adult Atlantic herring [37] and Atlantic mackerel (*Scomber scombrus*) [38], both of which would be detectable above our TS threshold. These fish are present in the area in high numbers in the summer and fall [15, 39], when the diel pattern in hourly fish counts was strongest. It is possible that the diffusion and formation of schools of fish, such as herring and mackerel, could have influenced the observed counts even if the number of fish present did not truly change. Ideally, schools would be included in this study, but to do so new processing techniques to separate schools of fish from entrained air need to be implemented [40].

Hydroacoustics could not reveal the species of fish at this site, but physical sampling that occurred from 2011 to 2014 [15, 41] indicated which species were likely present. These studies sampled the tidal channels of the bay with pelagic and benthic trawls and the intertidal areas with seine and fyke nets from spring to fall of each year. They found that Cobscook Bay holds a diverse fish assemblage, with 46 species sampled, many of which have seasonal in- and off-shore movements in the Gulf of Maine [42, 43]. Atlantic herring and winter flounder (*Pseudopleuronectes americanus*) were by far the most abundant species caught in the tidal channels, making up 59.6% and 27.1% of the catch [15]. Most fish sampled were juveniles, with lengths agreeing well with the TS of fish detected in this study. Some larger fish were also likely present and able to avoid the trawls, such as adult Atlantic mackerel in the summer and fall. Four diadromous species, which have well-defined annual on- and off-shore movements related to spawning, were also captured: alewife (*Alosa pseudoharengus*), rainbow smelt (*Osmerus mordax*), blueback herring (*Alosa aestivalis*), and American eel (*Anguilla rostrata*). The life stages of some of the fish species sampled were also found to change seasonally: herring sampled in May and June were typically larval, while those sampled in August and September were juvenile or adult. Some of the patterns we observed could have been related to fish growing into the size ranges we sampled by setting a -50 dB TS threshold. For example, while individual larval fish (not schooling) may be too weak to detect, juvenile fish several cm long would likely be strong enough targets. If larval fish, such as Atlantic herring, remain in Cobscook Bay and mature over the course of the spring and summer, their growth to lengths detectable above our threshold could contribute to the comparably higher hourly counts observed in summer and fall.

The changing patterns in fish counts that we observed each year are also likely related to the seasonally changing fish assemblage of Cobscook Bay. Vertical and horizontal movement patterns of fish in response to environmental cues are often specific to species and life stage [1, 44], and can change seasonally even for one species as fish respond to changing day length and temperature [11]. Though the vertical diel and tidal movements of most of the species present in Cobscook Bay are not well known, particularly in such fast flows, a seasonally shifting fish assemblage is likely to contribute to the seasonally shifting patterns in hourly counts that we observed. The presence of herring and mackerel in the summer and fall, for example, could have substantially contributed to the increase in night-time counts. Both species form schools that have been found to disperse under low light [37, 38], and Atlantic herring have been observed to carry out migrations into the water column at night [45, 46], either of which would bring more individuals into the sampled volume at night than during the day. Nightly school diffusion was evident in our previous surveys at the site sampling with a vertically-oriented echosounder [14], as were diel changes in vertical distribution, as previously discussed. Without the ability to separate acoustically detected fish by species, the unique movement pattern of any one species at this site is virtually impossible to separate from the combined movement patterns of all the others. In future studies of regions with diverse fish assemblages, it may be helpful to collect data with multiple acoustic frequencies to aid in distinguishing anatomically distinct groups of fish [47]. The uncertainty introduced to our analysis by the possibility of vertical migration could be reduced by using a vertically-facing bottom-mounted echosounder rather than a horizontally-oriented one. This would allow the characterization of temporal patterns in fish vertical distribution, as for nekton in [6, 19].

In the present study, we saw no consistent correlation between hourly fish counts and current speed, and this has implications for potential MHK device effects. Previous work in [48, 49] used video to observe fish interacting with MHK turbines at two other locations, and both studies concluded that fish were less abundant at high current speeds and therefore less likely to come in contact with moving turbine blades. Any consistent response to the tidal currents at our site would have appeared in the wavelet transform as a band near the 6-hr period, as current speed rises and falls every 6 hours (Fig 5b). This was not the case. Instead, we observed that fish counts were frequently high during the flowing tides (not only at slack tides), particularly at night from summer through fall. Many factors could contribute to this difference. For example, each of these three studies sampled different fish species and life stages (juvenile North Atlantic species, present study; adult pollack [48]; various adult reef fishes [49]), and each group could respond differently to fast currents. If hourly fish counts are assumed proportional to rate of encounter with an MHK turbine at the same depth, our results indicate that fish may be more likely to encounter moving turbine blades than might have been expected based on results of the previous studies. Additionally, previous studies were done with video, and so only sampled during the day. Our results indicated fish presence was often highest at night, when obstacle avoidance may be less likely [31, 50]. However, this speculation should be balanced with the results of laboratory studies of fish entrained in MHK turbines, which found survival rates generally exceeding 90%, at least under lit conditions [51, 52]. Even if the rate of fish encountering and entraining in an MHK turbine is high, the magnitude of direct turbine effects such as blade strike may still be minimal. This is particularly true for small fish, such as those detected here: as fish size relative to blade diameter decreases, injury and mortality have also been found to decrease [51]. More information is needed on the movements of fish near MHK devices in a field setting to assess the applicability of laboratory results to estimating the likelihood of entrainment and blade strike in the field.

Absence of a 6-hr periodicity in the wavelet transform also indicated no consistent relationship of fish detection probability with current speed. We previously observed that fish move more randomly during slack tides [31]. This is further supported by fish direction reported in this study (Fig 5). The number of fish counted in an hour at slack tide may therefore be inflated if the same individual is tracked multiple times because it passes through the beam more than once. Conversely, counts during the flowing tide could be conservative because fish may pass through the beam too quickly to be detected by sequential pings. Alternatively, the numbers could be inflated due to more water being sampled per unit time. The ping rate was sufficiently high relative to the current speed to make the former unlikely. Any of these results would be linked to current speed and would have a 6-hr periodicity. It is unlikely that they would occur in such perfect asynchrony as to cancel each other out for the entire 2 years of data collection. So, if detection probability varied with current speed, at some point in the time series, a 6-hr periodicity should have been evident. While the number of fish detected per unit time was the focus of this study, investigating counts adjusted for the amount of water sampled would be an interesting future investigation.

The high temporal variability in hourly fish counts has implications for monitoring the interactions of fish with MHK devices. For example, to monitor near-field interactions of fish with an MHK device (e.g., direct strike by turbine blades), it would be best to focus sampling efforts on times with high fish counts and a rotating turbine. At this site, the best time to observe turbine interactions in the winter and spring would likely be near sunrise or sunset during the second half of ebb tide, and in the summer and fall, at night during either flood or ebb tide. Consideration should be given to the types of fish (e.g. species and life stages) that would be present at different times, as they may all respond differently to an MHK device. For example, [49] noted differences in ‘boldness’ between the species interacting with the tidal turbine. Additionally, the natural movement and activity patterns of species at a particular location will influence when they are most likely to encounter a tidal turbine (e.g. at night vs. day, in fast vs. slow flow, as larvae vs. as adults), and therefore how they may respond.

One approach to quantifying less direct MHK device effects on fish is to monitor fish abundance in the area of a device before and during its deployment [31, 53]. It is important that results of such monitoring at tidal energy sites reflect actual trends in fish abundance: overestimating effects on fish could harm this developing renewable energy industry, but underestimating effects could harm the marine ecosystem. Unless sampling can be continuous, as in this study, the high cyclic variability in fish presence in such dynamic environments could influence results of a long-term study if it is not designed to consider these patterns. For example, if our echosounder were duty-cycled to collect data for just a few hours of the month (and therefore substantially reduce power usage and processing time), samples occurring at low tide would record higher numbers of fish relative to samples occurring at high tide, and this could generate patterns in the resulting time series that are not actually there (i.e., signal aliasing) [5]. On the other hand, knowledge of existing cycles could be used to improve monitoring techniques. For example, Urmy and Horne [19] limited their analysis of nekton vertical distribution to samples collected near noon, which reduced variation introduced by the diel vertical migration of organisms. At tidal energy sites, 24-hr surveys would be well-suited to characterizing the strong variation occurring over 12-hr and 24-hr cycles (and sometimes 6-hr cycles [48]). These surveys could then be spaced over time to characterize the longer-term cycles, such as seasonal changes. This would be a first step toward achieving accurate long-term monitoring results at tidal energy sites like Cobscook Bay. As more data are collected in the coming years, longer environmental cycles (e.g., climatic oscillations) will need to be taken into account, as they may also influence observed trends. This long-term variation would be difficult to characterize without studies spanning decades, but sampling a control or reference site alongside a tidal energy site would aid in separating this and other sources of variation (natural or anthropogenic) from effects of MHK devices.

Results presented here are specific to one part of the water column at one site, but they may be applicable to other tidal energy sites characterized by similarly high-magnitude environmental cycles. In strongly tidal channels frequented by valuable or endangered fish species (e.g., salmon in the northwest USA or Atlantic sturgeon in the northeast), observing fish presence at a device’s location with high temporal resolution could be very useful in predicting and mitigating MHK device effects. The numbers of fish at turbine depth could be combined with observations of close-range evasion behavior in the field, as well as fish injury and mortality rates obtained in laboratory settings, to estimate the effects of a single MHK device on the fishes sharing its depth. When continuous or high-frequency sampling is not an option, known environmental cycles should be used to inform plans for monitoring turbine effects on fish, as well as the interpretation of study results. Better understanding of the effects of a single MHK device on individual fish may help us predict the effects of devices on fish populations as this new renewable energy industry moves toward the deployment of commercial-scale MHK device arrays.

## Supporting information

S1 Fig Effects of data gap-filling methods on wavelet transform results. (TIF)
