# Supplementary figures and images for: Multi-scale temporal patterns in fish presence in a high-velocity tidal channel
Source: PLoS One. 2017 May 11;12(5):e0176405. doi: 10.1371/journal.pone.0176405 (PMC5426631; doi:10.1371/journal.pone.0176405)

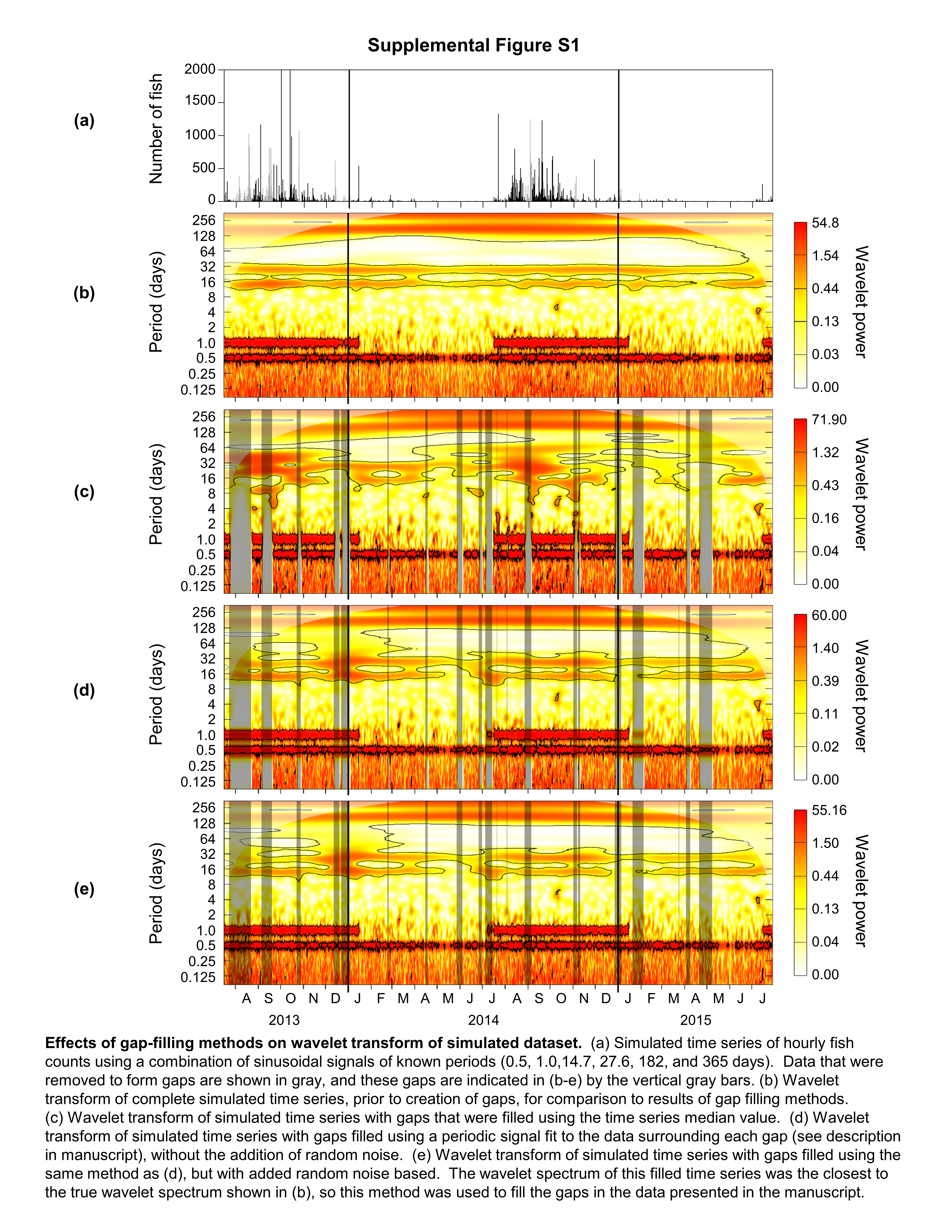

Supplement: S1 Fig — (TIF) [file pone.0176405.s001.tif]
